# Picroside II attenuates ischemia/reperfusion testicular injury by alleviating oxidative stress and apoptosis through reducing nitric oxide synthesis^1^

**DOI:** 10.1590/s0102-865020190110000002

**Published:** 2019-12-20

**Authors:** Yanze Li, Lei Wang, Zhiyuan Chen, Xiuheng Liu

**Affiliations:** I Master, Department of Urology, Renmin Hospital of Wuhan University, Wuhan, Hubei, P.R. China. Design of the study, technical procedures, interpretation of data, statistical analysis, manuscript preparation.; II MD, Department of Urology, Renmin Hospital of Wuhan University, Wuhan, Hubei, P.R. China. Design of the study, interpretation of data.; III Associate Professor, Department of Urology, Renmin Hospital of Wuhan University, Wuhan, Hubei, P.R. China. Conception and design of the study, critical revision.; IV Full Professor, Department of Urology, Renmin Hospital of Wuhan University, Wuhan, Hubei, P.R. China. Conception and design of the study, critical revision, final approval.

**Keywords:** Picroside, Ischemia, Reperfusion, Oxidative Stress, Apoptosis, Testis, Rats

## Abstract

**Purpose:**

To investigate the effect of Picroside II on testicular ischemia and reperfusion (l/R) injury and the underlying mechanism.

**Methods:**

Sprague-Dawley rats were randomly divided into 4 groups: sham operated group (Sham), Sham with Picroside II treatment group (Sham+ Pic II), l/R group (l/R) and l/R with Picroside II treatment group (I/R+ Pic II). l/R model was established by rotating the left testis 720° in a clock-wise direction for 4 hours. The histopathologic and spermatogenetic evaluation was performed. The apoptosis changes and the levels of HO-1 (heme oxygenase-1), MPO (myeloperoxidase), NOX (NADPH oxidase), SOD (superoxide dismutase), XO (xanthine oxidase) and NOS (nitric oxide synthase) were measured.

**Results:**

The seminiferous tubules were damaged in l/R rats, but Picroside II alleviated the changes induced by l/R. The increased level of apoptosis was decreased by Picroside II (P=0.01, 9.05±0.35 vs. 4.85±0.25). The activities of HO-1, MPO, NOX, XO and MDA content were increased and the SOD activity was decreased in l/R (P<0.05) and could be reversed by Picroside II (P=0.03, 405.5±7.5 vs. 304±17U/mgprot; P=0.02, 0.99±0.05 vs. 0.52±0.04 mgprot; P=0.01, 260+7 vs. 189±2 mgprot; P=0.04, 10.95+0.55 vs. 8.75+0.35 U/mgprot; P=0.045, 6.8+0.7 vs. 3.75+0.35 mgprot; P=0.04, 44.5+3.5 vs. 57.5+3.5 mgprot). Western blot showed that the expression of iNOS, nNOS and eNOS were increased in l/R (P<0.05); however, they were decreased after Picroside II treatment (P<0.05).

**Conclusion:**

Picroside II attenuated testicular I/R injury in rats mainly through suppressing apoptosis and oxidative stress through reduction of nitric oxide synthesis.

## Introduction

Testis torsion, a rotating of the spermatic cord and its contents, is a urology emergency. Annual incidence of spermatic cord torsion is 4.5 in 100.000 males 1-25 years of age^1^. If treated within 6 hours of the presenting pain, there is a good chance of saving the affected testicle, as 90%-100% testicles will be saved. If treated within 6 h-12 h, about 20%-50% testicles will be saved and if treated within 12 h-24 h, only 0%-10% testicles will be saved^2^. The degree of testis damage is dependent on the torsion degree and duration^3^. It was reported that survival rates of surgical detorsion was ranging from 42% to 88%, but the testicular function was unpredictable.

The pathophysiology of testis depended on ischemia/reperfusion (I/R) injury. And overgeneration of reactive oxygen species (ROS) plays a crucial role in I/R injury, causing the damage of testis spermatogenesis^4^. During reperfusion, ROS stimulated the generation and recruitment of inflammatory cytokines (such as interleukin-1β (IL-1β) and tumor necrosis factor-α (TNF-α), leading to testicular atrophy, germ cell apoptosis and destruction of spermatogenesis^5^ ^,^ ^6^.

Picroside II is a flavonoid compound extracted from a traditional Chinese herbal medicine, Picrorhiza scrophulariiflora pennell. Previously, it has been demonstrated that Picroside II possesses many effects, such as anti‐oxidative stress, anti-apoptosis and anti‐inflammation effects^7^ ^,^ ^8^. Picroside II has been applied to protect against I/R injury in many organs, including the brain, kidney and so on^9^ ^–^ ^11^. However, its effect on testis I/R has not been reported. In this study, we aimed to investigate the effects of Picroside II on testicular I/R injury and the underlying mechanism.

## Methods

All 24 adult male Sprague-Dawley rats (6-8 weeks, 220–250 g) were from the Center of Experimental Animals in Medical College, Wuhan University. The committee of experimental animals of Wuhan University approved this project, and the procedures carried out according to the routine animal-care guidelines.

All rats were housed at 20-24°C with 12 h light-12 h dark cycle and supplied with standard rat chow and water.

### I/R procedures

The rats in each group were anesthetized with pentobarbital (45 mg/kg) and placed on a homeothermic table to maintain core body temperature at 37 °C. All operations were performed under sterile conditions. Briefly, after anesthesia, a vertical paramedian incision of the scrotum was made and then unilateral testicular torsion was performed by rotating the left testis 720° in a clock-wise direction and fixed for 4 hours. At the end of 4 hours, the detorsion of the testis was performed after anesthesia and the testis was placed back into the scrotum^12^.

### Experimental protocol

All rats were randomly divided into four groups, each with six rats. Sham group was subjected to all operative procedures, except testis torsion and detorsion. Sham+ Pic II (Picroside II) group was the same as Sham group, together with administration of Picroside II (10 mg/kg). In I/R group, rats undergone 4 hours of testis torsion and 4 hours of detorsion. I/R+Pic II group was administrated with Picroside II (10 mg/kg) 30 minutes before testis detorsion, and the other procedures were the same with I/R group.

### Preservation of testis

The testis was removed under fully maintained anesthesia at the end of detorsion. After removal, the testis was fixed in 10% phosphate-buffered formalin, and stored at −80 °C for the following experiments.

### Intervention study

Picroside II (CAS No: 39012-20-9, purity >98%, molecular formula C23H28O13) was purchased from Tianjin Kuiqing Medical Technology Co. Ltd. We diluted it into 10g/L solution with 1 mol/L PBS. Picroside II (10 mg/kg) 250 μL was administrated via tail vein with a micro-syringe in Sham+ Pic II group and I/R+ Pic II group. And rats in I/R group and Sham group were injected 1 mol/L PBS 250 uL simultaneously.

### Hypothesis

After torsion/detorsion, testis structure will be destroyed, and the level of apoptosis will increase, and the level of oxidative stress will also ascent, and meanwhile the expression of NOS will grow. However, after the administration of Picroside II, all the changes will be reversed.

### Primary outcome measurements

Histologic examinations of testis tissues to observe the morphologic changes; TUNEL to learn the level of apoptosis; detection of some markers (like HO-1, SOD, NOX, etc.) to find the changes in oxidative stress; western blotting to test the expressions of nitric oxide synthesis.

### Secondary outcome measurement

The mean seminiferous tubule diameter (MSTD) and TUNEL index.

### Histologic examinations

The testis was fixed in 10% phosphate-buffered formalin, and then embedded with paraffin and sectioned at 4-μm thick. The sections were deparaffinized and hydrated, and stained with hematoxylin and eosin (H&E). Morphologic evaluations were performed by an experienced pathologist who didn't know the protocol. The mean seminiferous tubule diameter (MSTD) was measured and the Johnsen scores were evaluated according to Johnsen's Tubular Biopsy Scores (JTBS) and Table 1 ^13^.

**Table 1:** Histological criteria and the modified Johnsen score system for assessment of spermatogenesis.

Score	Histologic properties
10	Full spermatogenesis
9	Slightly impaired spermatogenesis, many late spermatids, disorganized epithelium
8	Less than five spermatozoa per tubule, few late spermatids
7	No spermatozoa, no late spermatids, many early spermatids
6	No spermatozoa, no late spermatids, few early spermatids
5	No spermatozoa or spermatids, many spermatocytes
4	No spermatozoa or spermatids, few spermatocytes
3	Spermatogonia only
2	No germinal cells, Sertoli cells only
1	No seminiferous epithelium

### TUNEL assay

Apoptotic level on paraffin sections of the testis was assessed by TUNEL (Terminal-deoxynucleotidyl-Transferase-mediated dUTP Nick End Labeling) with a commercial kit (Boehringer Mannheim, Mannheim, Germany). Control slides were incubated with 50μL Label solution and stained with 0.05% diaminobenzidine for 10 min. And then, we counterstained sections with Mayer's haematoxylin and observed them under a Nikon Eclipse 50i microscope at 400x magnification. Positive cells were marked by brown staining. The average number of TUNEL-positive cells per tubule was calculated in 10 random fields.

### Heme oxygenase-1 activity

Briefly, testis tissue was added to a reaction mixture. The reaction was performed at 37°C in the dark condition for 1 hour and then it was terminated by the addition of 1 mL of chloroform. The extracted bilirubin was calculated by the difference in absorbance between the wavelengths 464 and 530 nm.

### Nicotinamide adenine dinucleotide phosphate oxidase activity

Lucigenin-enhanced chemiluminescence was used to measure nicotinamide adenine dinucleotide phosphate (NADPH) oxidase activity in testis, according to the method described previously^14^.

### Measurement of Myeloperoxidase Activity

Activity of testicular myeloperoxidase (MPO), an enzyme that is found predominantly in the azurophilic granules of polymorphonuclear leukocytes, was measured by the method described previously^15^.

### Superoxide dismutase (SOD) and malondialdehyde (MDA) measurement

Commercial kits were used in accordance with the manufacturer's instructions (Nanjing Jiancheng Co., China) to measure SOD activity (xanthineoxidase method) and MDA concentration (thiobarbituric acid method).

### Assay for xanthine oxidase activity

Xanthine Oxidase Activity was measured by the method described previously^15^.

### Western Blot analysis

Total proteins were separated, and quantified by Bicinchoninic acid method. Then, equivalent weights of protein (40μg/lane) were separated on 10% SDS-PAGE gels and transferred to nitrocellulose membrane. 5% non-fat milk was used to block the membranes in TBST buffer. Then the membranes were incubated with primary antibodies against eNOS, nNOS, and iNOS (all obtained from Santa Cruz) at 4°C using slow rocking. Next, after washing twice with TBST, the membranes were blotted with secondary antibody, which was conjugated with horseradish peroxidase at 1:5000 dilution. Distinct bands were emphasized by using a chemiluminescence detection kit.

### Statistical analysis

The means of sample sizes were calculated in each group and data were presented as mean ± SEM. Student t and One-way ANOVA test with Bonferroni adjustment was used for comparing normally distributed data. Then the means of the different groups were compared by using one-way ANOVA and Student–Newman–Keuls test. When P<0.05, differences were considered statistically significant.

## Results

### Effect of Picroside II on testicular damage induced by I/R in rats

**Figure 1:**
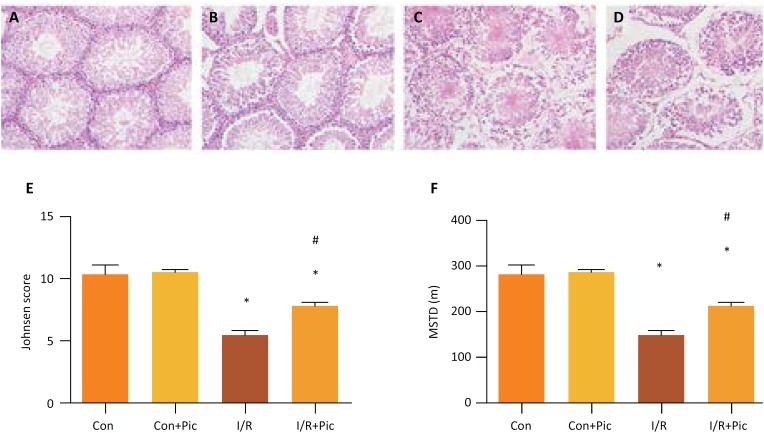
Effect of Picroside II treatment on testicular damage induced by ischemia/reperfusion (I/R) in rats. Paraffin-embedded testes sections were stained with hematoxylin and eosin. histopathological evaluation: **(A)** sham group, **(B)** sham+ Pic II group, **(C)** I/R group, **(D)** I/R +Pic II group. **(E, F)** Johnsen scores and quantification of mean seminiferous tubular diameter (MSTD) for each group. Values are means ± standard deviation, n=6 in each group. *P <0.05 *vs*. control group. #P <0.05 *vs.* I/R group.

The rats in the Sham group and Sham+ Pic II group both showed almost normal testis structure (Fig. 1A, B). And the rats in the I/R group showed destruction of tubular epithelium, necrosis, debris formation, congestion and atrophy (Fig. 1C). However, in I/R+ Pic II group, the testis structure was obviously improved (Fig. 1D).

According to Johnsen scoring system, the spermatogenesis in I/R group was reduced in comparison to the Sham and Sham+ Pic II group. Whereas, Picroside II treatment could reverse the reduction of spermatogenesis induced by testicular I/R (Fig. 1E). In addition, MSTD results showed that the Picroside II treatment had a larger MSTD than the I/R group (Fig. 1F).

### Effect of Picroside II treatment on testicular apoptosis induced by I/R in rats

**Figure 2:**
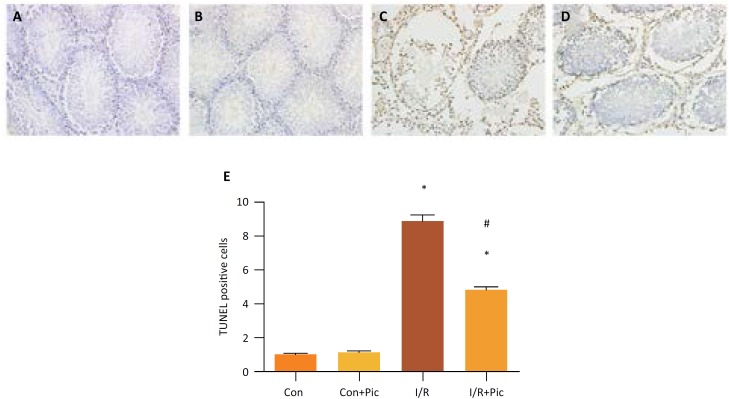
Effect of Picroside II treatment on testicular cells apoptosis induced by ischemia/reperfusion(I/R) injury in rats. **(A, B)** Sham group and Sham+ Pic II group with a few positive cells. **(C)** I/R group with a high level of positive cells. **(D)** I/R+ Pic II group with a lower level of positive cells than I/R group. **(E)** apoptosis index of each group. Values are means ± standard deviation, n=6 in each group. *P <0.05 *vs.* control group. #P <0.05 *vs.* I/R group.

TUNEL assay was used to investigate the apoptosis level induced by I/R^16^. Contrasted with Sham group and Sham+ Pic II group (Fig. 2 A,B), it had more TUNEL positive cells in I/R group (Fig. 2C). However, in I/R+ Pic II group, Picroside II treatment could inhibit cell apoptosis induced by testicular I/R (Fig. 2D). Apoptosis index was the quantification of TUNEL assay (Fig. 2E).

### Effect of Picroside II treatment on testicular oxidative stress (OS) induced by ischemia/reperfusion (I/R) injury in rats

The activities of HO-1, NOX, MPO, SOD, XO and content of MDA were detected to reflect rats testicular OS level induced by I/R. As the results showed, all of them had no difference between Sham group and Sham+ Pic II group (Table 2). However, the activities of HO-1, NOX, MPO, XO and content of MDA were downregulated in I/R group, when compared with Sham group and Sham+ Pic II group. Moreover, the treatment of Picroside II could improve the activities of HO-1, NOX, MPO, XO and content of MDA. Besides, we also found that Picroside II could reverse the decreased SOD activity induced by testicular I/R injury. All these results indicated that Picroside II could improve the oxidative stress induced by I/R in testis.

**Table 2:** Evaluation of oxidative stress markers for each group.

	Sham (n=6)	Sham+PicII (n=6)	I/R (n=6)	I/R+PicII (n=6)	P (ANOVA)
HO-1 activity (U/mgprot)	212±11	221±4	406±8	304±17	<0.05
NOX activity (U/mgprot)	107±6	108±1	260±7	189±2	< 0.05
MPO activity (U/mgprot)	0.18±0.06	0.16±0.05	0.99±0.05	0.52±0.04	< 0.05
SOD(U/mgprot)	74.5±4.5	77.5±1.5	44.5±3.5	57.5±3.5	<0.05
XO activity (U/mgprot)	6.00±0.10	5.95±0.05	10.95±0.55	8.75±0.35	<0.05
MDA (nmol/mgprot)	2.3±0.3	2.3±0.2	6.8±0.7	3.8±0.4	<0.05

The effect of Picroside II treatment on testicular oxidative stress (OS) induced by ischemia/reperfusion (I/R) injury in rats was evaluated. After 4-hour detorsion, testes were removed for determination of activities of heme oxygenase-1 (HO-1), NADPH oxidase (NOX), myeloperoxidase (MPO), superoxide dismutase (SOD), xanthine oxidase (XO) and malondialdehyde (MDA) content. Values are means ± standard deviation, n=6 in each group (Table 2).

### Effect of Picroside II treatment on testicular nitric oxide synthesis induced by ischemia/reperfusion (I/R) injury in rats

**Figure 3:**
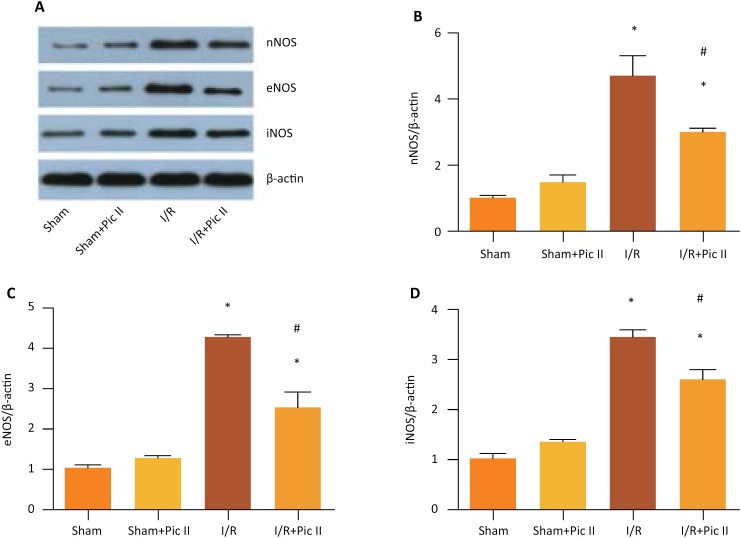
Effect of Picroside II treatment on testicular nitric oxide (NO) synthesis induced by ischemia/reperfusion (I/R) injury in rats. After 4-hour detorsion, Testis were removed for western blotting **(A)** to analyze protein expression of nNOS **(B)**, eNOS, **(C)** iNOS **(D)**. Values are means ± standard deviation, n =6 in each group. *P <0.05 *vs.* control group. #P <0.05 *vs.* I/R group.

Nitric oxide synthase (NOS), including three isoenzymes (nNOS, eNOS, iNOS), is the rate-limiting enzyme in the synthesis of nitric oxide, and its activity changes directly regulate the formation of nitric oxide and its biological effects^17^. Western blot results showed that the expression of nNOS, eNOS and iNOS had no difference between Sham group and Sham+ Pic II group. However, I/R could induce the expression of them and Picroside II treatment could reverse the increased expression induced by I/R (Fig. 3 A–D).

## Discussion

Testis torsion is an emergency in the urology department, usually in childhood or adolescence. Successful surgery for detorsion is necessary, but there are still 40%-80% patients with poor prognosis, including testicular atrophy and infertility. I/R injury in torsion/ detorsion is the direct and complex damage, including the significant increase of reactive oxygen radicals (ROS) and reactive nitrogen species (RNS)^18^. In our study, we performed testis torsion/detorsion as the model of testicular I/R injury.

Picroside II, a Chinese herb extract, has demonstrated pharmacological effects, such as antioxidant, anti-apoptotic, anti-inflammatory, anticarcinogenic, neuroprotective, hepatoprotective, anticholestatic effects, and immune modulating activities. Its effects are much stronger than those of other types of flavonoid. Due to its effects, Picroside II has proved to be effective on protecting against I/R injury, including heart, liver and kidney^9^ ^–^ ^11^. Although the effect of some other drugs hasbeen studied t on testicular I/R, there aren't many researches applying Chinese herb extracts, particularly Picroside II^19^ ^–^ ^22^.

It has been reported that testicular injury consists of structure destruction in morphology and spermatogenesis dysfunction. Previous studies showed that in I/R injury, testicular structure might change obviously, including testis weight decrease, atrophy, destruction of tubular epithelium, necrosis, debris formation and congestion^23^ ^,^ ^24^. In our study, the results showed the same. It is known that germ cells are the most sensitive to I/R injury, especially to lipid peroxidation, because there is a rich content of polyunsaturated fatty acids in their plasma membrane^25^ ^,^ ^26^. With the testis structure destruction, the function of spermatogenesis was also impaired, which was demonstrated based on Johnsen scoring system in our study^27^. However, the treatment of Picroside II could ameliorate testicular I/R injury, both in structure and function.

A previous study showed that testis I/R injury could produce large amount of ROS and inflammatory factors and enhance the spermatogenic cells apoptosis, leading to infertility^28^ ^–^ ^30^. In our study, we applied TUNEL assay to reveal the level of apoptosis induced by I/R. Our results were consistent with this previous study, as testis cells apoptosis level was increased after I/R; however, with Picroside II treatment, the apoptosis induced by I/R could be reduced.

I/R could cause oxidative stress by producing over production of reactive oxygen species (ROS), then leading to the destruction of lipids, proteins and DNA^31^ ^,^ ^32^. Based on this, we detected some markers to evaluate the changes of oxidative stress.

Heme oxygenase-1(HO-1) is a rate-limiting enzyme for heme degradation, and the degradation products have antioxidative function^33^. ‘It's been reported that HO-1 level increases in oxidative stress reaction^34^. NADPH oxidase (NOX) is a major source of ROS in a number of non-phagocytic cells, and its level changes indicate the variation of ROS^35^. Myeloperoxidase, MPO, is an important iron-containing lysosome. It's a functional marker and activation marker of neutrophils, and its level and activity changes represent the function and activity status of neutrophilic polymorphonuclear leukocytes (PMN)^36^. In our study, their level was increased after I/R; however, the treatment of Picroside II could significantly decrease the elevated level of them induced by I/R.

As we all know, superoxide dismutase (SOD) is an oxygen free radical scavenger, which is widely found in various tissues of organisms and can scavenge free radicals. And Malondialdehyde (MDA) is one of the most important products of membrane lipid peroxidation, which could be used to reflect the degree of membrane lipid peroxidation. Previous studies have shown that in testicular I/R injury, SOD activity is reduced and the content of MDA is increased, aggravating the damage of oxygen free radicals in testicular tissue^37^. Our results were consistent with previous results. SOD activity decreased and MDA content increased after I/R. However, treatment of Picroside II could obviously reverse the changed level induced by I/R. So, we consider that Picroside II could alleviate oxidative stress induced by testicular I/R injury.

Nitric oxide synthase (NOs) is the rate-limiting enzyme in the synthesis of nitric oxide, which is involved in the regulation of NO synthesis. It includes three different isoforms: endothelial NOS (eNOS), neuronal NOS (nNOS), and inducible NOS (iNOS). Our results showed that iNOS, eNOS, nNOS levels were increased after I/R injury and with the treatment of Picroside II, their upregulated levels were decreased. Therefore, we considered that Picroside II reduced NO synthesis to protect testis from I/R injury.

Comparing with the previous studies which used different kinds of treatments^19^ ^–^ ^22^, like nifedipine, urapidil and apocynin, we all got approximately the same results in the changes of testicular structures, the level of apoptosis and the level of some oxidative stress markers, including MDA and SOD. Nonetheless, there are also some differences between our studies. We detected more markers like HO-1, MPO, XO, and so on, and some of the previous studies detected some other markers like GPX, GSH, CAT, and so on. So, in a future study, we could use other makers for reference to do more research. Another thing that we observed is that we also analyzed the expression of three kinds of NOS respectively, indicating the role of NO synthesis in the testicular I/R injury^19^ ^–^ ^22^.

Overall, we found that Picroside II could protect against testicular I/R injury and might be a potential therapeutic agent in the future. But there were still some limitations in our study. The first one is that the sample size was calculated, as we referred to some articles and used “6 rats” in each group. But the results were measured by calculating the mean rats in each group. The second one is that, as some reports have showed that NO has double-edged eﬀects in I/R injury, which is dependent on the tissue, site, source, and environment, in our next research, we will investigate more about NO effects in testis I/R injury. Finally, the third one was that there was no experiment in vitro; so, next time we will need to conduct an in vitro experiment.

## Conclusion

Picroside II attenuated testicular I/R injury in rats, mainly through suppressing apoptosis and oxidative stress, and the mechanism was probably through reduction of nitric oxide synthesis.
